# The influence of the interval between percutaneous transhepatic gallbladder drainage and cholecystectomy on perioperative outcomes: a retrospective study

**DOI:** 10.1186/s12876-021-01810-9

**Published:** 2021-05-19

**Authors:** Koichi Kimura, Eisuke Adachi, Sachie Omori, Ayako Toyohara, Takahiro Higashi, Kippei Ohgaki, Shuhei Ito, Shin-ichiro Maehara, Toshihiko Nakamura, Yoichi Ikeda, Yoshihiko Maehara

**Affiliations:** Department of Surgery, Kyushu Central Hospital of the Mutual Aid Association of Public School Teachers, 3-23-1, Shiobaru, Minamiku, Fukuoka City, 815-8588 Japan

**Keywords:** Acute cholecystitis, PTGBD, Cholecystectomy

## Abstract

**Background:**

Percutaneous transhepatic gallbladder drainage (PTGBD) is recommended for acute cholecystitis patients at high risk for surgical treatment. However, there is no evidence about the best timing of surgery after PTGBD. Here, we retrospectively investigated the influence of the interval between PTGBD and surgery on perioperative outcomes and examined the optimal timing of surgery after PTGBD.

**Methods:**

We performed a retrospective analysis of 22 patients who underwent cholecystectomy after PTGBD from January 2008 to August 2019. We examined perioperative factors between patients with an interval of ≤ 7 days between PTGBD and cholecystectomy (≤ 7-day group; n = 12) and those with an interval of ≥ 8 days (≥ 8-day group; n = 10). Moreover, we also examined perioperative factors between patients with an interval of ≤ 14 days from PTGBD to cholecystectomy (≤ 14-day group; n = 10) and those with an interval of ≥ 15 days (≥ 15-day group; n = 12).

**Results:**

Of the 22 patients, 9 had Grade I cholecystitis, 12 had Grade II cholecystitis, and 2 had Grade III cholecystitis. Nine patients had high-grade cholecystitis before PTGBD and 13 had a poor general condition. We examined perioperative factors between patients with an interval of ≤ 7 days between PTGBD and cholecystectomy (≤ 7-day group; n = 12) and those with an interval of ≥ 8 days (≥ 8-day group; n = 10). The C-reactive protein (CRP) level before surgery was significantly higher (12.70 ± 1.95 mg/dL vs. 1.13 ± 2.13 mg/dL, *p* = 0.0007) and the total hospitalization was shorter (17.6 ± 8.0 days vs. 54.1 ± 8.8 days, *p* = 0.0060) in the ≤ 7-day group than in the ≥ 8-day group. We also examined perioperative factors between patients with an interval of ≤ 14 days from PTGBD to cholecystectomy (≤ 14-day group; n = 14) and those with an interval of ≥ 15 days (≥ 15-day group; n = 8). The CRP level before surgery was significantly higher (11.13 ± 2.00 mg/dL vs. 0.99 ± 2.64 mg/dL, *p* = 0.0062) and the total hospitalization was shorter (19.5 ± 7.2 days vs. 59.9 ± 9.5 days, *p* = 0.0029) in the ≤ 14-day group than in the ≥ 15-day group. However, there were no significant differences between the ≤ 14-day group and the ≥ 15-day group in the levels of hepatic enzymes before surgery, adhesion grade, amount of bleeding during surgery, operative duration, frequency of surgical complications, or length of hospitalization after surgery.

**Conclusions:**

The interval between PTGBD and surgery has little influence on perioperative outcomes.

## Background

Acute cholecystitis (AC) is a common surgical emergency worldwide [1]. Although the treatment guidelines for AC are well-established by the Tokyo Guidelines 2018 and the Tokyo Guidelines 2013 [2], some points are still unclear. Minimally invasive procedures, including endoscopic and percutaneous techniques, are preferred for gallbladder drainage of AC, and percutaneous transhepatic gallbladder drainage (PTGBD) is recommended for patients with AC with a poor general condition [3, 4]. PTGBD is useful for improving the general condition of patients with AC at a high risk for surgery; however, PTGBD is only conservative treatment, and surgery should still be performed. PTGBD cannot completely treat AC, and many patients develop recurrent cholecystitis [5–7].

Several studies have discussed the optimal interval between PTGBD and surgery; however, the findings are inconsistent [8–11]. Some reports found that a shorter interval between PTGBD and surgery resulted in shorter hospital stays and lower medical costs than a longer interval, with comparable intraoperative and postoperative outcomes [8]. Conversely, some reports determined that the interval between PTGBD and surgery was not correlated with the duration of anesthesia or postoperative hospital stay [10]. Lyu et al. reported that the timing of cholecystectomy after PTGBD does not affect surgical complications, although performing surgery as soon as possible after PTGBD could decrease hospital stay and reduce medical costs. On the other hand, some have reported that a shorter interval between PTGBD and surgery was associated with more frequent postoperative complications. Fujinaga et al. reported that cholecystectomy after PTGBD was associated with a longer operation time, more intraoperative blood loss, more conversion to laparotomy from laparoscopic surgery, and a higher frequency of surgical complications [11]. Moreover, Won et al. suggested that early laparoscopic cholecystectomy is feasible following PTGBD, especially in patients with low risk [12]. Therefore, the optimal timing for surgery after PTGBD is still unclear.

## Methods

In the current study, we retrospectively investigated the influence of the interval between PTGBD and surgery on perioperative outcomes and examined the optimal timing of surgery after PTGBD.

## Patients

**Fig. 1:**
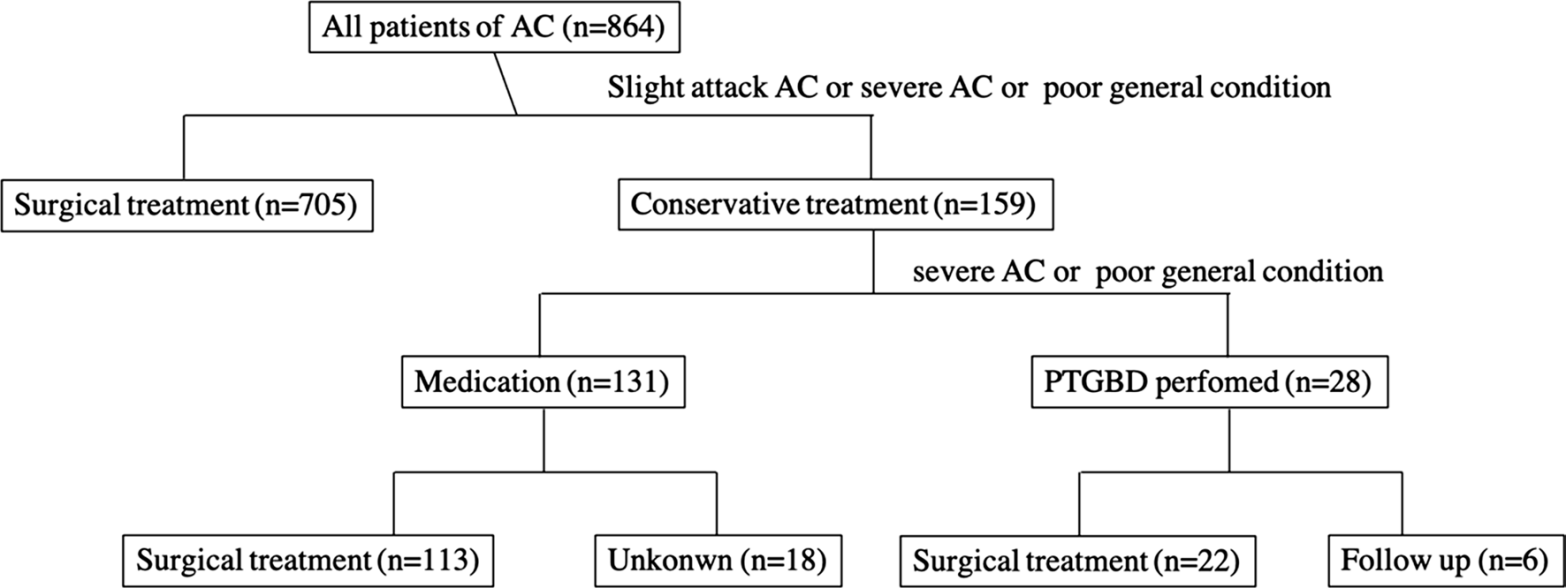
Patient’s treatment process

A total of 22 adults (> 20 years old) who underwent PTGBD before surgery for AC at the Kyushu Central Hospital from January 2008 to December 2019 were included in this study. All treatment procedures were performed after obtaining full informed consent from the patients. Medical charts were retrospectively reviewed to obtain the patients’ data. Patient’s treatment process are described Fig. 1.

## Cholecystitis diagnosis and severity assessment

We conformed to the Tokyo Guidelines 2018 diagnostic criteria and severity assessment criteria for AC [13]. The diagnostic criteria for AC were as follows: local signs of inflammation such as Murphy’s sign or right upper abdominal quadrant mass/pain/tenderness; systemic signs of inflammation such as fever, elevated C-reactive protein (CRP), and elevated white blood cell count (WBC); and imaging findings characteristic of AC. The combination of local inflammation/upper abdominal symptoms and systemic inflammation indicated a suspected diagnosis of AC, and these symptoms plus imaging findings indicated a definite diagnosis. Grade III AC was associated with cardiovascular dysfunction (hypotension requiring treatment with dopamine ≥ 5 µg/kg per min or any dose of norepinephrine), neurological dysfunction (decreased level of consciousness), respiratory dysfunction (PaO_2_/FiO2 ratio < 3004), renal dysfunction (oliguria or creatinine > 2.0 mg/dL), hepatic dysfunction (PT-INR > 1.5), or hematological dysfunction (platelet count < 100,000/mm^3^). Grade II AC was associated with elevated WBC count (> 18,000/mm^3^), palpable tender masses in the right upper abdominal quadrant, duration of complaints > 72 h, or marked local inflammation (gangrenous cholecystitis, pericholecystic abscess, hepatic abscess, biliary peritonitis, or emphysematous cholecystitis). Grade I AC was AC not meeting the criteria of Grade III or Grade II AC. It could also be defined as AC in a healthy patient with no organ dysfunction and mild inflammatory changes in the gallbladder, making cholecystectomy a safe and low-risk operative procedure.

## Adhesion grade

We referred to the adhesion grade described by Suzuki et al. [14], as follows: Grade 0: slight adhesion; Grade 1: adhesion localized in just one field of vision; Grade 2: more widespread adhesion than that of Grade 1, e.g., in the intestine and abdominal wall, gastroepiploic artery and intestine, or abdominal wall; and Grade 3: adhesion in the whole intraperitoneal space, making adhesiotomy impossible.

### Statistical analysis

All values are expressed as means and standard deviations. Categorical variables were compared using χ^2^ tests. *P* < 0.05 was considered statistically significant. All statistical analyses were performed using JMP software (SAS Institute Japan, Tokyo, Japan).

## Results

### Patient characteristics

Twenty-two patients underwent cholecystectomy after PTGBD between January 2008 and August 2019. The mean age of the patients was 76 ± 10 years. The comorbidities and cholecystitis grade have been described in Table 1).

**Table 1:** Patient background characteristics

Factors	Patients (n = 22)
Age (years), mean ± standard deviation	76 ± 10
Gender (Male, %)	14 (63.6)
Comorbidity	18 (81.8)
Diabetes mellitus (%)	8 (36.4)
Hypertension (%)	7 (31.8)
Ischemic heart disease (%)	5 (22.7)
Past laparotomy (%)	2 (9.1)
Respiratory disease (%)	2 (9.1)
Cerebrovascular disease (%)	2 (9.1)
Chronic hepatitis (%)	2 (9.1)
Steroid medication (%)	2 (9.1)
Dementia (%)	1 (4.5)
Anticoagulant medication (%)	2 (9.1)
Cholecystitis grade	
Grade I (%)	8 (36.4)
Grade II (%)	12 (54.5)
Grade III (%)	2 (9.1)

Data are presented as n (%) unless otherwise indicated

### Reasons for PTGBD

Nine patients (40.9 %) underwent PTGBD as the first treatment procedure before surgery because of high-grade cholecystitis, and 13 (59.1 %) had a poor general condition. The mean interval from a crisis of cholecystitis to PTGBD was 3 ± 7.1 days. No patients experienced complications of PTGBD. The mean WBC count before PTGBD was 13,025 ± 6,603 /mL, and the CRP level before PTGBD was 18 ± 10.54 mg/dL (Table 2).

**Table 2:** Reasons for PTGBD

Factors	Patients (n = 22)
Reason for PTGBD as the first procedure	
High-grade cholecystitis, n (%)	9 (40.9)
Poor general condition, n (%)	13 (59.1)
Time from crisis to PTGBD (days), mean ± standard deviation	3 ± 7.1
PTGBD-related complications, n (%)	0 (0.0)
WBC count before PTGBD (/mL), mean ± standard deviation	13,025 ± 6603
CRP before PTGBD (mg/dL), mean ± standard deviation	18 ± 10.54

PTGBD: percutaneous transhepatic gallbladder drainage, WBC: white blood cell, CRP: C-reactive protein

### Outcomes of surgery

The mean interval between PTGBD and surgery was 7 ± 38.6 days. The mean WBC count before surgery was 8,550 ± 5,642/mL, and the mean CRP before surgery was 3 ± 8.84 mg/dL. The American Society of Anesthesiologists physical statuses were one in 2 patients (9.1 %), two in 10 patients (45.5 %), and three in 10 patients (45.5 %). The adhesion grades were Grade 0 in 2 patients (9.1 %), Grade 1 in 4 patients (18.2 %), Grade 2 in 14 patients (63.6 %), and Grade 3 in 2 patients (9.1 %). The mean operating time was 124 ± 39 min, and the mean blood loss during surgery was 50 ± 235 g. Seven patients (31.8 %) converted to laparotomy surgery from laparoscopic surgery. Two patients (9.1 %) developed surgical complications: one had bleeding after surgery and required reoperation for hemostasis, and one had an intraperitoneal abscess after surgery and required antibiotic medication. The mean length of hospitalization after surgery was 17 ± 19.2 days, and the mean total hospitalization was 29 ± 32.9 days (Table 3).

**Table 3:** Outcomes of surgery

Factors	Patients (n = 22)
Interval from PTGBD to surgery (days), mean ± standard deviation	7 ± 38.6
WBC count before surgery (/mL), mean ± standard deviation	8550 ± 5642
CRP before surgery (mg/dL), mean ± standard deviation	3 ± 8.84
ASA score	
1	2 (9.1)
2	10 (45.5)
3	10 (45.5)
Adhesion grade	
Grade 0	2 (9.1)
Grade 1	4 (18.2)
Grade 2	14 (63.6)
Grade 3	2 (9.1)
Operating time (min), mean ± standard deviation	124 ± 39
Blood loss (g), mean ± standard deviation	50 ± 235
Laparotomy conversion	7 (31.8)
Surgical complication	2 (9.1)
Bleeding after surgery	1 (4.5)
Intraperitoneal abscess	1 (4.5)
Hospitalization after surgery (days), mean ± standard deviation	17 ± 19.2
Total hospitalization (days), mean ± standard deviation	29 ± 32.9

Data are presented as n (%) unless otherwise indicated

PTGBD: percutaneous transhepatic gallbladder drainage, WBC: white blood cell, CRP: C-reactive protein, ASA: American Society of Anesthesiologists

### The influence of the interval from PTGBD to surgery on patient outcomes

We next investigated the influence of the interval between PTGBD and surgery on patient outcomes. We compared the outcomes between patients with an interval of ≤ 7 days (≤ 7-day group; n = 12) and those with an interval of ≥ 8 days (≥ 8-day group; n = 10) (Table 4). The ≤ 7-day group had significantly less comorbidity than the ≥ 8-day group (*p* < 0.0181). Further, the CRP before surgery was significantly higher in the ≤ 7-day group than in the ≥ 8-day group (*p* < 0.0007), and the total hospitalization time was significantly shorter in the ≤ 7-day group than in the ≥ 8-day group (*p* < 0.0060).

**Table 4:** Comparison between patients undergoing surgery at ≤ 7 and ≥ 7 days following PTGBD

Factors	≤ 7-day group (n = 12)	≥ 8-day group (n = 10)	*P*
Gender (Male), n (%)	7 (58.3)	7 (70.0)	0.5696
Age (years)	74.8 ± 2.9	77.8 ± 3.1	0.4794
**Comorbidity (Yes), n (%)**	**8 (66.7)**	**10 (100.0)**	**0.0181**
Anticoagulant medication (Yes), n (%)	1 (8.3)	1 (10.0)	0.8925
ASA status	2.33 ± 0.19	2.40 ± 0.21	0.8195
Cholecystitis grade	1.9 ± 0.2	1.5 ± 0.2	0.1257
WBC before PTBGD (/mL)	13,355 ± 1952	13,892 ± 2138	0.8549
CRP before PTGBD (mg/dL)	19.38 ± 3.01	14.06 ± 3.30	0.2480
WBC before surgery (/mL)	10,064 ± 1638	7948 ± 1795	0.3942
**CRP before surgery (mg/dL)**	**12.70 ± 1.95**	**1.13 ± 2.13**	**0.0007**
Surgical complications (Yes), n (%)	1 (8.3)	1 (10.0)	0.8925
Operating time (min)	113.4 ± 11.3	131.0 ± 12.4	0.3062
Blood loss (g)	171.3 ± 69.2	133.8 ± 75.8	0.7185
Adhesion grade	1.6 ± 0.2	1.9 ± 0.2	0.3475
Laparotomy conversion (Yes), n (%)	4 (33.3)	3 (30.0)	0.8671
Hospitalization after surgery (days)	12.0 ± 5.4	24.2 ± 5.9	0.1406
**Total hospitalization (days)**	**17.6 ± 8.0**	**54.1 ± 8.8**	**0.0060**

Bold values indicate significant differences

Data are presented as mean ± standard deviation unless otherwise indicated

≤ 7-day group: patients with an interval between PTGBD and surgery ≤ 7 days, ≥ 8-day group: patients with an interval between PTGBD and surgery ≥ 8 days, PTGBD: percutaneous transhepatic gallbladder drainage, WBC: white blood cell, CRP: C-reactive protein, ASA: American Society of Anesthesiologists

We also compared the outcomes between patients with an interval between PTGBD and surgery of ≤ 14 days (≤ 14-day group; n = 10) and those with an interval of ≥ 15 days (≥ 15-day group; n = 12) (Table 5). The ≤ 14-day group had significantly less comorbidity than the ≥ 15-day group (*p* < 0.0426). Further, the CRP before surgery was significantly higher in the ≤ 14-day group than in the ≥ 15-day group (*p* < 0.0007), and the total hospitalization was significantly shorter (*p* < 0.0060)

We further compared the outcomes using 3-day and 5-day intervals, but there were no significant differences between groups in these comparisons

**Table 5:** Comparison between patients undergoing surgery at < 14 and > 14 days following PTGBD

Factors	≤ 14-day group (n = 10)	≥ 15-day group (n = 12) *P*	*P*
Gender (Male), n (%)	9 (64.3)	5 (62.5)	0.9333
Age (years)	75.4 ± 2.7	77.5 ± 3.5	0.6324
**Comorbidity (Yes), n (%)**	**10 (71.4)**	**8 (100.0)**	**0.0426**
Anticoagulant medication (Yes), n (%)	2 (14.3)	0 (0.0)	0.1658
ASA status	2.36 ± 0.18	2.38 ± 0.24	0.9529
Cholecystitis grade	1.9 ± 0.2	1.5 ± 0.2	0.2092
WBC before PTBGD (/mL)	13,141 ± 1800	14,402 ± 2382	0.6771
CRP before PTGBD (mg/dL)	18.19 ± 2.85	14.80 ± 3.77	0.4807
WBC before surgery (/mL)	10,191 ± 1492	7198 ± 1973	0.2404
**CRP before surgery (mg/dL)**	**11.13** ± **2.00**	**0.99** ± **2.64**	**0.0007**
Surgical complications (Yes), n (%)	1 (7.1)	1 (12.5)	0.6795
Operating time (min)	117.4 ± 10.6	128.5 ± 14.1	0.5345
Blood loss (g)	176.1 ± 63.8	116.1 ± 84.4	0.5772
Adhesion grade	1.6 ± 0.2	1.9 ± 0.3	0.5082
Laparotomy conversion (Yes), n (%)	5 (35.7)	2 (25.0)	0.5999
Hospitalization after surgery (days)	12.8 ± 4.9	25.9 ± 6.5	0.1259
**Total hospitalization (days)**	**19.5** ± **7.2**	**59.9** ± **9.5**	**0.0060**

Bold values indicate significant differences

Data are presented as mean ± standard deviation unless otherwise indicated

≤ 14-day group: patients with an interval between PTGBD and surgery ≤ 14 days, ≥ 15-day group: patients with an interval between PTGBD and surgery ≥ 15 days, PTGBD: percutaneous transhepatic gallbladder drainage, WBC: white blood cell, CRP: C-reactive protein, ASA: American Society of Anesthesiologists

## Discussion

We revealed that patients with a longer interval between PTGBD and cholecystectomy had more comorbidities than those with a shorter interval when examining intervals of both 7 days and 14 days, but there was no significant difference when examining intervals of 3 days or 5 days. Patients were considered for surgical treatment after PTGBD after confirmation of improvement of physical findings and inflammatory findings. Because patients with comorbidities show delayed improvement in general condition, they may need a longer interval from PTGBD to surgery.

We also found that the CRP level before surgery was significantly higher in patients with a shorter interval between PTGBD and cholecystectomy in both comparisons (7-day and 14-day). However, there was no difference between the two groups in the frequency of surgical complications, operating time, amount of blood loss during surgery, adhesion grade, or the rate of laparotomy conversion. The Tokyo Guidelines 2013 recommend that surgical treatment for cholecystitis be performed within 72 h from onset [15–17]; however, the Tokyo Guidelines 2018 suggest surgery be performed as soon as possible after onset and give no time limit [2]. These changes have improved surgical outcomes, including those of laparoscopic surgery. In our study, there was no significant difference in the surgical outcomes between patients based on the interval between PTGBD and cholecystectomy. However, these results may depend on patient backgrounds.

The total hospitalization of patients with a shorter interval between PTGBD and cholecystectomy was significantly shorter than that of patients with a longer interval. However, this result is attributable to the shorter interval between PTGBD and surgery; there was no difference in the length of hospitalization after surgery between the two groups. Therefore, surgical treatment should be performed as soon as possible for the efficiency of turnover of hospitalized patients and lower medical costs.

Some limitations of this analysis should be mentioned. First, we selected patients from one center. A multicenter study with a larger number of patients and greater variation in surgical techniques would help us reach more definitive conclusions. Second, this was a retrospective study and might be subject to investigative bias.

## **Conclusions**

There was no difference in perioperative risks and surgical outcomes between patients with a shorter interval between PTGBD and cholecystectomy and those with a longer interval. Rapid surgery after PTGBD could reduce total hospitalization and medical costs.

## Data Availability

The datasets analyzed during the current study are available from the corresponding author on reasonable request.

## Abbreviations

AC: acute cholecystitis
PTGBD: percutaneous transhepatic gallbladder drainage
WBC: white blood cell
CRP: C-reactive protein
